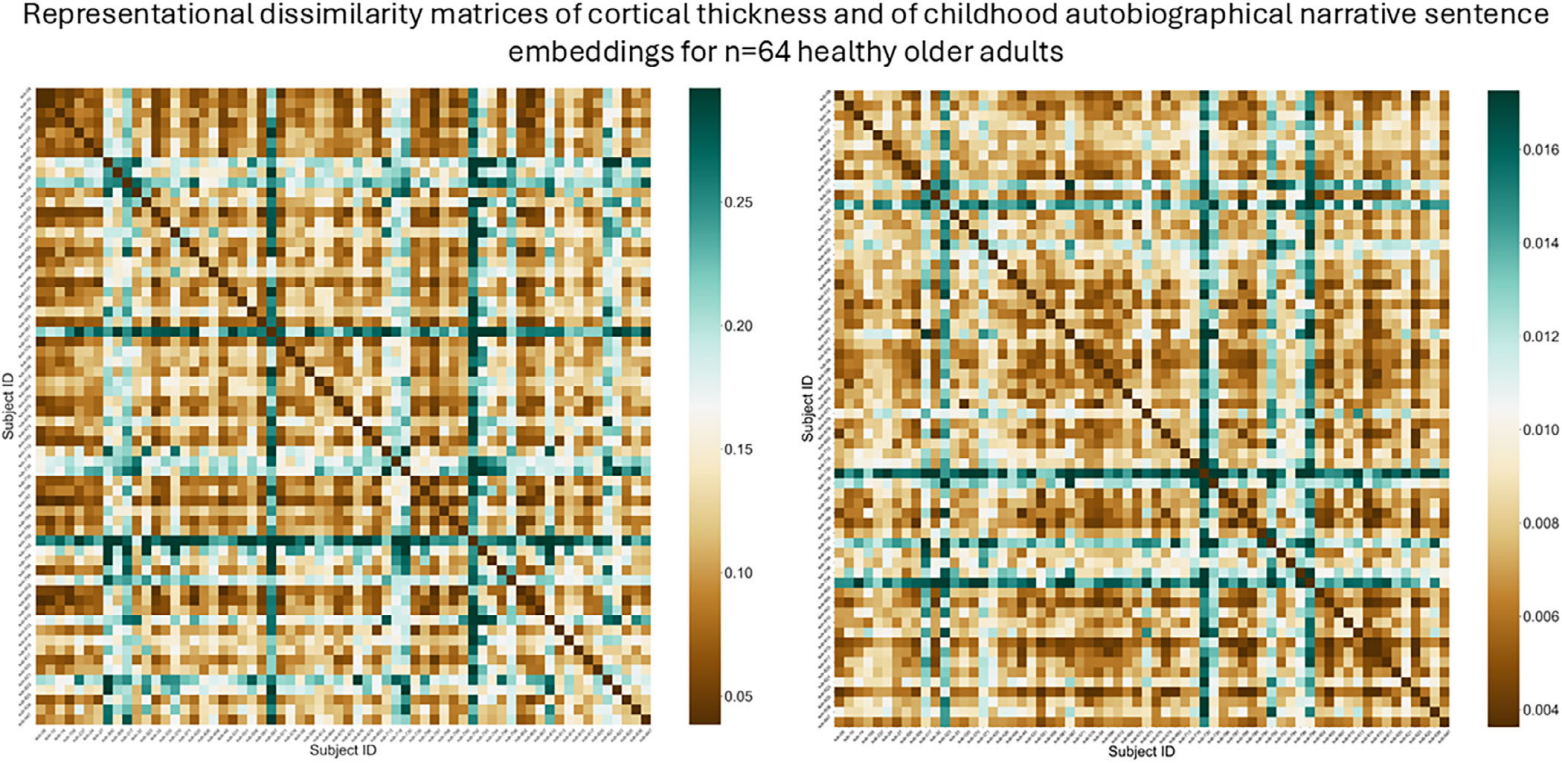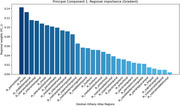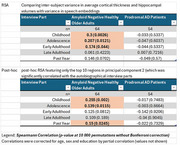# Brain‐language coupling: a representational similarity analysis of autobiographical speech and cortical morphometry in healthy aging and prodromal Alzheimer's disease

**DOI:** 10.1002/alz70861_108732

**Published:** 2025-12-23

**Authors:** Kevin Statz, Helena Balabin, Laure Spruyt, Bastiaan Tamm, Stefan Sunaert, Patrick Dupont, Rik Vandenberghe, Laboratory for Cognitive Neurology, Leuven Brain Institute

**Affiliations:** ^1^ KU Leuven, Leuven Belgium; ^2^ University Hospitals Leuven, Leuven Belgium

## Abstract

**Background:**

Automated natural language processing (NLP) of connected speech is a potential biomarker for incipient Alzheimer’s disease (AD). We previously demonstrated that NLP analysis of autobiographical narratives provides the highest specificity for distinguishing prodromal AD from amyloid‐negative healthy older adults. Here we determine the underlying neuroanatomical basis.

**Method:**

We analyzed automatically transcribed autobiographical interviews from 118 Flemish‐speaking individuals —64 amyloid‐PET–negative healthy controls and 54 biomarker‐proven prodromal AD patients—covering five life periods (childhood, adolescence, early adulthood, late adulthood, past year). A transformer model generated 768‐dimensional embeddings per interview period. We also extracted cortical thicknesses of 68 Desikan–Killiany (34 per hemisphere) and intracranial‐volume‐adjusted left and right hippocampal volumes from T1‐weighted MRIs using FreeSurfer. Representational similarity analysis (RSA) was used to determine Spearman correlations between subject‐wise dissimilarity matrices based on speech embeddings and on brain morphometry controlling for age, sex and education through partial correlation. Significance was evaluated by permutation testing with Bonferroni correction. Principal‐component analysis (PCA) on cortical thickness identified regions driving inter‐subject variance.

**Result:**

In healthy amyloid‐negative older adults, between‐subject similarity of embeddings from childhood, adolescence and early‐adulthood narratives significantly correlated with the between‐subject similarity in brain morphometry (Spearman ρ = 0.300, 0.207, 0.174; *p* =0.00026, 0.012, 0.044, resp.). This was mainly driven by the correlation between cortical thickness and embeddings from narrative (Spearman ρ = 0.300, *p* =0.013). The second PCA component (encompassing mostly bilateral pars orbitalis, lateral orbitofrontal, insular and inferior temporal cortices and the right middle temporal cortex) significantly correlated with the speech embeddings. Among these regions.

**Conclusion:**

In cognitively healthy, amyloid‐negative older adults, the semantic structure of autobiographical memory partly relates to orbitofrontal, inferior frontal and lateral temporal cortical thickness patterns, with a temporal gradient. This coupling is lost in prodromal AD, likely due to neurodegeneration of lateral temporal regions implicated in autobiographical memory.